# Cocaine in the Incoercible Vomitings of Pregnancy

**Published:** 1887-04

**Authors:** 


Cocaine in the Incoercible Vomitings of Pregnancy.—Cocaine has lately come into use for the incoercible vomitings of pregnancy, and in several cases reported by Weiss, Englemann, Holtz and Bois, it seems to have given good results. Weiss prescribes a teaspoonful every half hour of a solution containing fifteen centigrammes of hydrochlorate of cocaine in one hundred and fifty grammes of water. Englemann and Holtz use a three per cent. solution in ten to thirty drop doses, while Bois applies to the neck of the uterus, night and morning, a pomatum in which one centigramme of cocaine is incorporated with fifty grammes of vaseline. Fraipont prefers the hypodermic method, injecting under the skin a Pravaz syringe full of a four per cent. solution, and claims signal success in other forms of obstinate vomiting as well as in the vomitings of pregnancy.—*Boston Med. and Surg. Jour.*

---

A GOOD way to give turpentine is in the following emulsion, published in *The Doctor:*

| | |
|---|---|
| Oil of turpentine, - - - | 2 ounces. |
| White of egg, - - - - | 2 " |
| Glycerine, - - - - | 4 " |
| Syrup, - - - - - | 4 " |
| Water, - - - - - | 4 " |

Mix the white of egg and glycerine together, add the oil of turpentine and shake thoroughly; then add the syrup and, lastly, the water, shaking them well together. This makes a nice emulsion, and is easily made and as permanent as any turpentine emulsion. A teaspoonful dose will contain about eight minims of turpentine.

---

A NORWEGIAN doctor, H. J. Vetlesen, of Hamar, gives some hundred and sixteen cases of whooping-cough, in which he obtained favorable results in over seventy-one per cent. of the cases, and entire cures in twenty-five per cent., with the use of the following mixture:

| | | |
|---|---|---|
| ℞ Ext. cannabis indicæ, - - | 1 gramme. | |
| Ext. belladonnæ, - - - | 50 centigrammes. | |
| Alcoholis, (90°,) - - - | 5 grammes. | |
| Glycerini, - - - - - | 5 grammes. | M. |

S. Give four to twenty drops morning and evening, according to the age of the child. Dr. Vetlesen tried the extracts separately, but could not obtain the same results as when they were combined as above.—*Phila. Med. Times.*

